# Supplementary material for: Hepatic Resection Following Selective Internal Radiation Therapy for Colorectal Cancer Metastases in the FOXFIRE Clinical Trial: Clinical Outcomes and Distribution of Microspheres
Source: Cancers (Basel). 2019 Aug 12;11(8):1155. doi: 10.3390/cancers11081155 (PMC6721483; doi:10.3390/cancers11081155)
Supplement: Supplementary file 1 [file cancers-11-01155-s001.pdf]

Supplementary Materials: Hepatic Resection Following Selective Internal Radiation Therapy for Colorectal Cancer Metastases in the FOXFIRE Clinical Trial: Clinical Outcomes and Distribution of Microspheres

Helen Winter, Joseph Rassam, Pradeep S. Virdee, Rob Goldin, Priyankaa Pitcheshwar, Klara Weaver, John Primrose, David P. Berry, Harpreet S. Wasan and Ricky A. Sharma

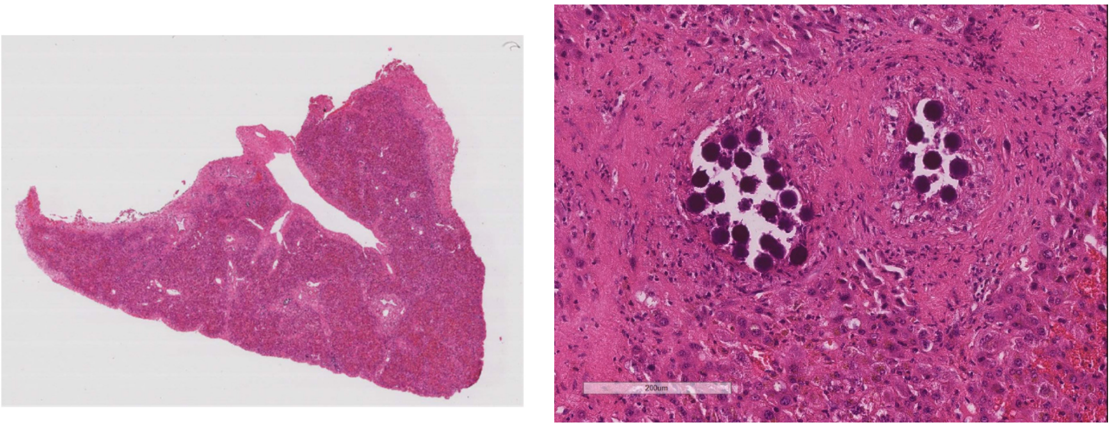

(A) **(B)**

Figure S1. (**A**) Low power view of liver, with microspheres taken from a digitised image (Haematoxylin and Eosin stained section, original magnification ×20). (**B**) High power view of microspheres taken from the same digitised image as the previous slide (Haematoxylin and Eosin stained section, original magnification ×400).

Table S1. Microsphere densities and distribution demonstrated on zonal analysis. Spheres/mm^2 in 0-1mm and 1-2mm zones away from tumour border, 0-1mm and 1-2mm zones within tumour border, non-neoplastic tissue and tumour centre. Tumour centre was defined as area proximal to 2mm within tumour border. n/a = tissue not available.

Cases	Spheres/mm^2 1–2 mm zone away from tumour	Spheres/mm^2 0–1 mm zone away from tumour	Spheres/mm^2 0–1 mm zone into tumour	Spheres/mm^2 1–2 mm zone into tumour	Spheres/mm^2 Non-neoplastic tissue	Spheres/mm^2 Tumour centre
**Case 1**	1.25	2.96	3.33	0.48	0.57	1.49
**Case 2**	0.95	3.19	15.94	0.88	0.25	1.23
**Case 3**	1.51	3.47	12.6	8.87	0.55	1.69
**Case 4**	1.58	2.26	2.16	3.21	0	1.32
**Case 5**	1.01	1.95	3.72	0.72	4.09	0
**Case 6**	2	2.31	2.38	2.01	0.74	1.53
**Case 7**	1.87	3.61	24.72	18.74	0	2.5
**Case 8**	1.12	2.1	2.07	0.25	0.18	0.33
**Case 9**	0	0.09	0.13	0	0.11	n/a
**Case 10**	0	0	0.05	0	0	0.22
**Case 11**	0	0	0	0.14	n/a	0.16
**Median**	**1.12**	**2.26**	**2.38**	**0.72**	**0.215**	**1.28**
**IQR**	**1.58**	**3.1**	**12.47**	**3.07**	**0.57**	**1.31**

Table S2. Histological findings in tumour and non-neoplastic liver tissue in individual cases receiving chemotherapy-alone or chemotherapy plus SIRT. Abbreviation used: n/a= tissue not available.

Case Number	Tumour Tissue					Non-Neoplastic Tissue Properties		
	Viable Tumour	Necrosis	Fibrosis	Inflammation/ Macrophage Infiltration	Other Properties	Vascular Change	Nodular Regenerative Hyperplasia	Steatosis
**Chemo + SIRT cases**								
Case 1	0%	20%	60%	20%	No	Moderate	Yes	No
Case 2	0%	0%	90%	10%	Evidence of calcification	Mild	No	No
Case 3	0%	50%	20%	0%	30% Mucinous	Moderate	Yes	No
Case 4	0%	0%	70%	20%	10% Mucinous	Severe	No	No
Case 5	0%	0%	80%	20%	No	Severe	No	No
Case 6	10%	0%	70%	20%	No	n/a	n/a	n/a
Case 7	0%	0%	50%	50%	No	Mild	No	No
Case 8	10%	0%	20%	0%	70% Mucinous	Moderate	No	No
Case 9	80%	10%	10%	0%	No	Mild	No	Mild
Case 10	50%	5%	35%	5%	No	Mild	No	No
Case 11	50%	50%	0%	0%	No	n/a	n/a	n/a
**Chemo alone cases**								
Case 12	n/a	n/a	n/a	n/a	n/a	Mild	Yes	No
Case 13	80%	10%	10%	0%	No	Mild	Yes	No
Case 14	50%	30%	20%	0%	No	Moderate	No	No
Case 15	10%	75%	5%	10%	No	n/a	n/a	n/a
Case 16	80%	0%	0%	20%	No	Mild	No	No
Case 17	n/a	n/a	n/a	n/a	n/a	Moderate	Yes	No
Case 18	85%	0%	15%	0%	No	Moderate	No	No
Case 19	60%	30%	10%	0%	No	Moderate	No	No
Case 20	60%	40%	0%	0%	No	Moderate	No	No
Case 21	0%	100%	0%	0%	No	Mild	No	No
Case 22	10%	50%	40%	0%	No	Mild	No	Mild
Case 23	n/a	n/a	n/a	n/a	n/a	Mild	No	Mild
Case 24	20%	60%	10%	0%	No	Moderate	No	No
Case 25	60%	20%	20%	0%	No	Moderate	No	Mild
Case 26	0%	30%	40%	0%	30% Mucinous	Moderate	No	No
Case 27	20%	0%	60%	20%	No	Moderate	Yes	No

Table S3. Histological effects seen in hepatic resection specimens after chemotherapy. Abbreviations used: FOLFOX: fluorouracil/leucovorin, oxaliplatin; FOLFIRI: fluorouracil/leucovorin, irinotecan; FOLFOXIRI: fluorouracil/leucovorin, oxaliplatin and irinotecan; 5-FU: 5-fluorouracil; LV: leucovorin; HAI: hepatic arterial infusion; NASH: non-alcoholic steato-hepatitis; SOS: sinusoidal obstruction syndrome.

Author	Prospective or Retrospective	Number of Patients	Systemic Therapy	Histological Effects	Observations
(Rubbia-Brandt et al., 2004) [1]	Retrospective	153	FOLFOX, 5-FU, FOLFIRI, FOLFOXIRI	51% sinusoidal dilatation and haemorrhage 78% receiving oxaliplatin had sinusoidal alterations	Sinusoidal obstruction particularly related to oxaliplatin
(Vauthey et al., 2006) [2]	Retrospective	406: 158 no chemo vs 248 chemo	FOLFIRI, FOLFOX, 5-FU, other	9% steatosis; 8% steatohepatitis; 5% sinusoidal dilation	Irinotecan associated with steatohepatitis, associated with increased mortality at 90 days

(Aloia et al., 2006) [3]	Retrospective	92, 17 no chemo vs 75 chemo	FOLFOX, 5-FU/LV	52% chemo had vascular lesions vs 18% with no chemo	Main lesions from FOLFOX vascular Risk of complications related to duration of pre-operative chemo
(Fernandez et al.)2005) [4]	Prospective	37: 13 no chemo; 10 5FU; 14 irinotecan or oxaliplatin (4 both)	5-FU; FOLFOX; FOLFIRI; FOLFOXIRI		Graded for NASH & by liver injury score.
(Vreuls et al., 2016) [5]	Retrospective	30 resections; 28 had neoadjuvant oxaliplatin	oxaliplatin based chemotherapy	Showed detachment of the sinusoidal endothelial cell lining and an enlarged space of Disse.	Eighteen (64%) of the 28 patients showed SOS lesions, based on microscopy.
(Lu et al., 2013) [6]	Retrospective	106: 53 no chemotherapy; 42 neoadjuvant oxaliplatin (few had irinotecan); 11 had HAI	oxaliplatin 31% (& irinotecan 9%)	16 patients (15%) had steatosis, 31 29% had sinusoidal dilation and 19% had steatohepatitis. HAI more steatosis, sinusoidal dilation and steatohepatitis.	Oxaliplatin associated with sinusoidal dilation.

Table S4. Histological changes observed in hepatic resection specimens from patients having SIRT prior to resection. HCC: hepatocellular carcinoma. CRCLM: colorectal cancer liver metastases.

Publication	N	Diagnosis	Background Liver	Tumour Effects	Microsphere Distribution	Observations	Gallbladder Involvement
(Kennedy, A. S. et al., 2004) [7]	4	2 HCC 2 CRCLM	Little radiation effect on normal liver parenchyma, distant to tumour	The two patients with CRCLM showed 90% necrosis in all tumour nodules	Periphery of tumour (more than centre or background normal liver parenchyma)	Come centrilobular necrosis. Mild steatosis and the endothelium showed features of injury.	Two cases of cholecystitis – one with microspheres in submucosa
(Wang et al., 2013) [8]	3	CRCLM	Sinusoidal dilatation / tumour mass effect in adjacent liver	In five tumours, range of response from complete to minimal response on pathological assessment. Tumour necrosis, mucinous alteration, aggregates of foamy histiocytes, calcification and fibrosis.	Vascular tumour bed Vessels of portal tracts of background liver	Giant cell reaction or histiocytes Stromal fibrinous exudate with fibroblasts and delicate collagen strands, within the tumour bed. Minimal cellular inflammatory response; this observation is supportive of direct radiation injury as a non-immune mediated process.	Bile ductular reaction adjacent to tumour
(Justinger et al., 2015) [9]	13	CRC	Portal fibrosis in normal liver parenchyma adjacent to tumour at 10 weeks	Tumour regression with central scarring	Resin microspheres within vascular tumour bed.	Microspheres present in portal tract vessels in background liver	Not reported

(Hadaki et al., 2011) [10]	1	CRC	Microspheres trapped within the background parenchyma	Two discrete areas of fibrosis with a small amount of extracellular mucin, but no epithelial component confirming complete pathological resolution.	Trapped within the background parenchyma	Not reported	Not reported

References

1. Rubbia-Brandt, L.; Audard, V.; Sartoretti, P.; Roth, A.D.; Brezault, C.; Le Charpentier, M.; Dousset, B.; Morel, P.; Soubrane, O.; Chaussade, S., et al. Severe hepatic sinusoidal obstruction associated with oxaliplatin-based chemotherapy in patients with metastatic colorectal cancer. *Ann. Oncol.* **2004**, *15*, 460–466, doi:10.1093/annonc/mdh095.
2. Vauthey, J.N.; Pawlik, T.M.; Ribero, D.; Wu, T.T.; Zorzi, D.; Hoff, P.M.; Xiong, H.Q.; Eng, C.; Lauwers, G.Y.; Mino-Kenudson, M., et al. Chemotherapy regimen predicts steatohepatitis and an increase in 90-day mortality after surgery for hepatic colorectal metastases. *J. Clin. Oncol.* **2006**, *24*, 2065–2072, doi:10.1200/JCO.2005.05.3074.
3. Aloia, T.; Sebagh, M.; Plasse, M.; Karam, V.; Levi, F.; Giacchetti, S.; Azoulay, D.; Bismuth, H.; Castaing, D.; Adam, R. Liver histology and surgical outcomes after preoperative chemotherapy with fluorouracil plus oxaliplatin in colorectal cancer liver metastases. *J. Clin. Oncol.* **2006**, *24*, 4983–4990, doi:10.1200/JCO.2006.05.8156.
4. Fernandez, F.G.; Ritter, J.; Goodwin, J.W.; Linehan, D.C.; Hawkins, W.G.; Strasberg, S.M. Effect of steatohepatitis associated with irinotecan or oxaliplatin pretreatment on resectability of hepatic colorectal metastases. *J. Am. Coll. Surg.* **2005**, *200*, 845–853, doi:10.1016/j.jamcollsurg.2005.01.024.
5. Vreuls, C.P.; Driessen, A.; Olde Damink, S.W.; Koek, G.H.; Duimel, H.; van den Broek, M.A.; Dejong, C.H.; Braet, F.; Wisse, E. Sinusoidal obstruction syndrome (SOS): A light and electron microscopy study in human liver. *Micron* **2016**, *84*, 17–22, doi:10.1016/j.micron.2016.02.006.
6. Lu, Q.Y.; Zhao, A.L.; Deng, W.; Li, Z.W.; Shen, L. Hepatic histopathology and postoperative outcome after preoperative chemotherapy for Chinese patients with colorectal liver metastases. *World J. Gastrointest Surg.* **2013**, *5*, 30–36, doi:10.4240/wjgs.v5.i3.30.
7. Kennedy, A.S.; Nutting, C.; Coldwell, D.; Gaiser, J.; Drachenberg, C. Pathologic response and microdosimetry of (90)Y microspheres in man: review of four explanted whole livers. *Int. J. Radiat. Oncol. Biol. Phys.* **2004**, *60*, 1552–1563, doi:10.1016/j.ijrobp.2004.09.004.
8. Wang, L.M.; Jani, A.R.; Hill, E.J.; Sharma, R.A. Anatomical basis and histopathological changes resulting from selective internal radiotherapy for liver metastases. *J. Clin. Pathol.* **2013**, *66*, 205–211, doi:10.1136/jclinpath-2012-201231.
9. Justinger, C.; Kouladouros, K.; Gartner, D.; Tatsch, K.; Reimer, P.; Rudiger, T.; Binnenhei, M.; Bentz, M.; Schon, M.R. Liver resection after selective internal radiotherapy (SIRT): Proof of concept, initial survival, and safety. *J. Surg. Oncol.* **2015**, *112*, 436–442, doi:10.1002/jso.24000.
10. Hadaki, M.; Praseedom, R.; Brais, R.; See, T.C.; Balan, K.; Wilson, C.B. Selective internal radiation therapy with 90Y-SIR-Spheres microspheres for non-resectable colorectal metastases in the liver. *BMJ Case Rep.* **2011**, *2011*, doi:10.1136/bcr.01.2011.3793.

© 2019 by the authors. Licensee MDPI, Basel, Switzerland. This article is an open access article distributed under the terms and conditions of the Creative Commons Attribution (CC BY) license (http://creativecommons.org/licenses/by/4.0/).
